# Prognostic Value of Post-PCI Angiography-Derived Fractional Flow Reserve: A Systematic Review and Meta-Analysis of Cohort Studies

**DOI:** 10.3390/jpm13081251

**Published:** 2023-08-12

**Authors:** Dimitrios Terentes-Printzios, Konstantia-Paraskevi Gkini, Dimitrios Oikonomou, Vasiliki Gardikioti, Konstantinos Aznaouridis, Ioanna Dima, Konstantinos Tsioufis, Charalambos Vlachopoulos

**Affiliations:** First Department of Cardiology, National and Kapodistrian University of Athens, Medical School, Hippokration Hospital, 114 Vasilissis Sofias St., 11527 Athens, Greece; gk.konstantia@gmail.com (K.-P.G.); jimoik4@hotmail.com (D.O.); gardikiotiv@gmail.com (V.G.); conazna@yahoo.com (K.A.); ioadim2006@yahoo.gr (I.D.); kptsioufis@gmail.com (K.T.); cvlachop@otenet.gr (C.V.)

**Keywords:** angiography-derived FFR, percutaneous coronary intervention, QFR, quantitative flow ratio, vFFR

## Abstract

The post-percutaneous coronary intervention (post-PCI) fractional flow reserve (FFR) can detect suboptimal PCI or residual ischemia and potentially lead to fewer adverse clinical outcomes. We sought to investigate the predictive value of the angiography-derived FFR for adverse cardiovascular events in patients after PCI. We conducted a comprehensive search of electronic databases, MEDLINE, EMBASE, and the Cochrane Library, for studies published until March 2023 that investigated the prognostic role of angiography-derived fractional flow reserve values after PCI. We investigated the best predictive ability of the post-PCI angiography-derived FFR and relative risk (RR) estimates with 95% confidence intervals (CIs) between post-PCI angiography-derived FFR values and adverse events. Thirteen cohort studies involving 6961 patients (9719 vascular lesions; mean follow-up: 2.2 years) were included in this meta-analysis. The pooled HR of the studies using specific cut-off points for post-PCI angiography-derived FFR was 4.13 (95% CI, 2.92–5.82) for total cardiovascular events, while the pooled HRs for target vessel revascularization, cardiac death, target vessel myocardial infarction, and target lesion revascularization were 6.87 (95% CI, 4.93–9.56), 6.17 (95% CI, 3.52–10.80), 3.98 (95% CI, 2.37–6.66) and 6.27 (95% CI, 3.08–12.79), respectively. In a sensitivity analysis of three studies with 1789 patients assessing the predictive role of the post-PCI angiography-derived FFR as a continuous variable, we found a 58% risk reduction for future adverse events per 0.1 increase in the post-PCI angiography-derived FFR value. In conclusion, post-PCI angiography-derived FFR is an effective tool for predicting adverse cardiovascular events and could be potentially used in decision making, both during PCI and in the long-term follow-up.

## 1. Introduction

Coronary artery disease (CAD) is a leading cause of death worldwide. Specifically, 126 million individuals globally suffer from ischemic heart disease (1655 per 100,000), representing approximately 2% of the worldwide population in 2017 [1]. Percutaneous coronary intervention (PCI), depending on the specific clinical setting in which it is performed (in acute coronary syndrome or chronic coronary syndromes), has been shown improve quality of life and prognosis [2,3] In most large-scale trials, physiology-guided PCI using a pressure wire to assess the fractional flow reserve (FFR) has been shown to be superior to angiography-only guided PCI and is thus currently advocated by guidelines for decision making during PCI [4,5,6]. Despite the benefit of FFR-guided revascularization, the increased time and cost of the physiology assessment result in its underutilization, according to real-world data [7]. As a result, a wealth of new software assessing the angiography-derived FFR have emerged.

The angiography-derived FFR can assess the hemodynamic severity of coronary stenosis by combining fluid dynamics computation and a 3D anatomical vessel reconstruction based on angiographical views without the involvement of coronary vessel instrumentation with a pressure wire and the administration of vasodilator agents. One method, the quantitative flow ratio (QFR), has shown good correlation and diagnostic accuracy compared to the FFR [7,8,9]. Recently, FAVOR III China described that QFR-guided PCI was associated with improved 1- and 2-year clinical outcomes compared to the standard coronary angiography-guided PCI [10,11].

Although both PCI techniques and equipment have rapidly evolved in recent years, a significant proportion of patients undergoing an angiographically successful PCI suffer from adverse events, such as recurrent angina or silent ischemia [12]. A post-PCI functional assessment can detect suboptimal PCI or residual ischemia, leading to possible efforts to optimize the final result to reduce further the risk of adverse clinical outcomes. The immediate post-stenting measurement of the FFR could aid in optimizing revascularization results and potentially improve outcomes as it is well established that suboptimal PCI is an independent predictor of major cardiac adverse events [13,14,15]. Recent studies demonstrated the role of the post-PCI angiography-derived FFR as a predictor of a vessel- or patient-oriented outcomes in patients with stable CAD, acute coronary syndromes, or in-stent restenosis [16,17,18,19,20,21,22,23,24,25,26,27,28]. The primary objective of this meta-analysis was to evaluate whether the post-PCI angiography-derived FFR predicts coronary adverse events in CAD patients undergoing PCI. Second, we sought to investigate whether publication bias could have affected our results. Third, we evaluated the effects of several demographic and angiographic factors on the possible predictive role of the post-PCI angiography-derived FFR to identify the phenotype of the patient that would benefit most from such an assessment.

## 2. Materials and Methods

The systematic review and meta-analysis were conducted in accordance with the PRISMA 2020 checklist [29] (see also Supplementary Material). The outcomes of interest were: (1) total CV events (including vessel-oriented composite endpoint (VOCE), defined as the composite of cardiac death, vessel-related myocardial infarction (MI), or ischemia-driven target vessel revascularization (TVR) or major adverse cardiovascular events (MACE) or target lesion failure (TLF)), (2) TVR, (3) cardiac death, (4) target vessel MI and (5) TLR (target lesion revascularization). TLR was defined as revascularization post-stenting within the stent or within the 5 mm borders adjacent to the stent.

### 2.1. Data Sources and Research

For this systematic review and meta-analysis, a systematic search of the literature was performed in the PubMed, Cochrane, and Embase databases for cohort studies published until March 2023 that investigated the prognostic role of the post-PCI angiography-derived FFR. The following search terms were used: QFR, quantitative flow ratio, quantitative flow ratio AND coronary artery disease, QFR AND coronary artery disease AND (POST AND QFR AND prognosis, QFR AND prognosis, POST AND quantitative flow ratio AND prognosis, angiography-derived FFR. angiography-derived Fractional Flow Reverse AND vFFR. The search was not restricted to any language. Data sources were also identified by manually searching the references of articles, reviews and meta-analyses. We subsequently searched online resources such as the abstracts for major cardiovascular conventions and clinicaltrials.gov (accessed on 4 May 2023) to ensure the identification of all published and unpublished studies.

### 2.2. Study Selection

Studies were considered eligible if they met the following criteria: (1) were full-length publications in peer-reviewed journals; (2) were randomized-controlled studies, case studies, or cohort studies, either retrospective or prospective; (3) included patients with CAD who underwent PCI; (4) recorded the post-PCI angiography-derived FFR value; (5) reported a VOCE, defined as the composite of cardiac death, vessel-related MI, or TVR or MACE or TLR; and (6) had a minimum follow-up period of up to 6 months. No restriction criteria were imposed regarding the size of the population studied or the type of the population (chronic or acute coronary syndromes).

### 2.3. Data Extraction and Quality Assessment

Two reviewers (K.-P.G. and D.O.) independently conducted the data extraction, study selection and evaluation for the risk bias of the studies. Disagreements were resolved via consensus. The same 2 reviewers independently extracted data regarding the study population, intervention types, sample size, mean age, gender, follow-up period, indications of procedures, method of assessing the angiography-derived FFR, the cut-off point of the angiography-derived FFR and the studies’ outcomes and results. The quality of each study was evaluated via the Newcastle–Ottawa Scale (NOS) [30]. The NOS scale evaluates the quality of research by assessing the study population, comparability and outcome. This scale allocates up to 9 points for the lowest risk of bias in 4 domains: the selection of study groups (4 points), the comparability of the groups (2 points) and the ascertainment of exposure and outcomes (3 points). A study’s quality was considered poor if its Newcastle–Ottawa score was below 7.

### 2.4. Data Synthesis and Analysis

Each study described the risk estimates as hazard ratios (HRs), relative risks (RRs), odds ratios or dichotomous frequency data. We managed HRs as RRs. Fully adjusted RRs were selected over crude estimates, whenever available, as provided by the authors in multivariable regression models. We investigated the prognostic value of the post-PCI angiography-derived FFR by extracting and pooling RRs for the following outcomes from each study: (1) VOCE, including cardiac death, vessel-related MI and ischemia-driven TVR; (2) MACE; and (3) TLR. Moderate to significant heterogeneity existed among the studies, and a random-effects model was subsequently implemented. To test whether the true effect in all studies was the same (i.e., heterogeneity), we used the I-squared measure (I^2^), which permits the quantification of discrepancy among studies. Forest plots were created for a graphical representation of the individual studies’ RRs and confidence intervals (CIs).

We also performed a sensitivity analysis of three studies in which the RR for the post-PCI angiography-derived FFR were described as a continuous variable and calculated the adjusted, pooled RR per 0.1 increase in the post-PCI angiography-derived FFR value for total cardiovascular events.

The contribution of continuous study moderators to the overall heterogeneity was assessed via a meta-regression analysis with fixed-effects estimates. Publication bias was illustrated graphically via funnel plots, and its associations with our results were evaluated via the Duval and Tweedie trim-and-fill method and the classic fail-safe N method, as introduced by Rosenthal.

All analyses were performed with comprehensive meta-analysis version 2 (Biostat, Englewood, NJ, USA). We deemed statistical significance to be *p* < 0.05.

## 3. Results

### 3.1. Literature Search Results

**Figure 1:**
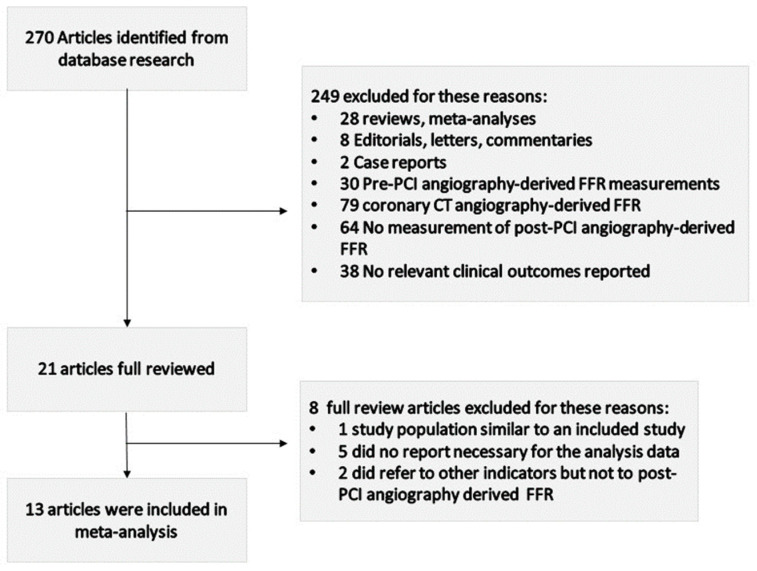
Flow diagram of study selection procedure.

Our initial systematic search of the literature retrieved 270 studies, 13 of which were suitable for the analysis (Figure 1). In total, 249 articles were excluded from this meta-analysis after reading the titles and the abstracts because they were irrelevant to the research purpose. Specifically, 28 studies were systematic review articles, 8 studies were editorials, letters or commentaries, 2 studies were case reports, 30 studies reported the pre-PCI angiography-derived FFR, 79 studies investigated the coronary CT angiography-derived FFR, 64 studies had no measurement of the post-PCI angiography-derived FFR and 38 studies had no relevant clinical outcome reported. Finally, eight articles were excluded after a full review for the following reasons: one study had a population similar to an included study [31], five studies did not report the data necessary for this analysis [16,32,33,34,35] and two did refer to other indicators but not to the post-PCI angiography-derived FFR [36,37].

### 3.2. Study Characteristics

Our meta-analysis included 13 original articles published since 2019. The included studies investigated 6961 patients (9719 vascular lesions; mean follow-up: 2.2 years). Several populations, such as patients with chronic ischemic heart disease, acute coronary syndrome and in-stent restenosis after PCI-DES, were contained in this meta-analysis. For the analysis of the total coronary adverse events, all but one study reported the HR without using a cut-off point [21]. Details of the individual studies regarding the association of the angiography-derived FFR with coronary artery events are provided in Table 1. The sample sizes ranged from 169 to 1805 individuals. Almost all studies examined age, sex and other cardiovascular risk factors.

### 3.3. The Effect of the Post-PCI Angiography-Derived FFR on Total Cardiovascular Events

**Figure 2:**
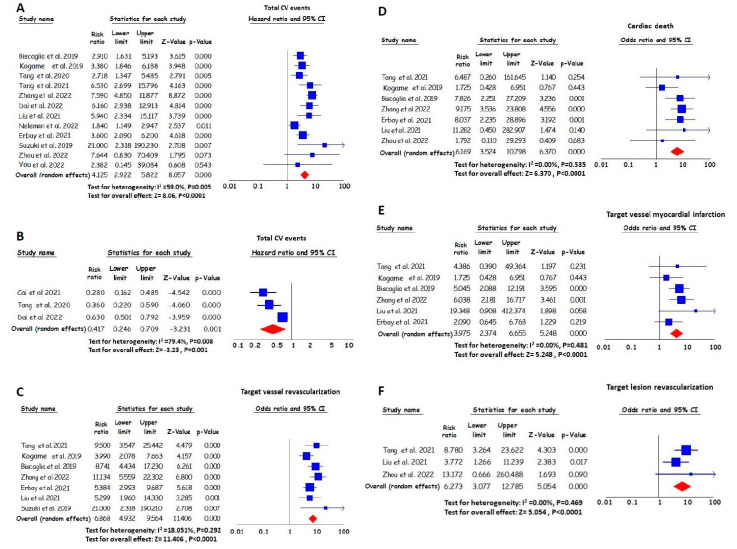
RR and 95% CI for lower angiography-derived FFR values and total cardiovascular (CV) events ((**A**) based on cut-off values [16,17,18,19,20,22,23,24,25,26,27,28]; (**B**) per 0.1 increase [19,20,21]), target vessel revascularization (**C**) [16,18,22,23,24,25,26], cardiac death (**D)** [16,18,22,23,24,25,28], target vessel myocardial infarction (**E**) [16,18,22,23,24,25] and target lesion revascularization (**F**) [16,22,28]. The squares’ sizes show the weight of each study, and the lines illustrate the 95% CI for individual studies with a lower and upper limit. The diamonds and their width represent the combined results of the meta-analysis.

The magnitude of risk for cardiovascular events in subjects with post-PCI angiography-derived FFR values below the cut-off provided by each study (12 studies in total, in which 11 studies reported VOCE and 1 study MACE) was significantly higher compared with the risk of individuals with higher post-PCI angiography-derived FFR values. Patients with lower angiography-derived FFR values after PCI experienced a four times higher risk of cardiovascular events during the follow-up period. Specifically, the total RR value was 4.13 (95% CI, 2.92–5.82) (Figure 2A).

In three studies, the post-PCI angiography-derived FFR value was considered a continuous variable. An increase per 0.1 in the pooled post-PCI angiography-derived FFR value resulted in a 58% reduction in the risk of cardiovascular events (HR 0.42 [95% CI, 0.25–0.71], per 0.1 increase) (Figure 2B).

### 3.4. The Effect of the Post-PCI Angiography-Derived FFR on TVR

The magnitude of risk for TVR in individuals with lower post-PCI angiography-derived FFR values was significantly higher compared with the magnitude of risk for individuals with higher values. Specifically, patients with lower post-PCI angiography-derived FFR values had an approximately seven times greater pooled RR for TVR during the follow-up period. The RR value was 6.87 (95% CI, 4.93–9.56) (Figure 2C).

### 3.5. The Effect of the Post-PCI Angiography-Derived FFR on Cardiac Death

Patients with lower post-PCI angiography-derived FFR values experienced higher levels of risk for cardiac death compared to those with higher post-PCI angiography-derived FFR values. Specifically, the pooled RR value was 6.17 (95% CI, 3.52–10.80) (Figure 2D).

### 3.6. The Effect of the Post-PCI Angiography-Derived FFR on Target Vessel MI

The magnitude of risk for target vessel MI in individuals with lower post-PCI angiography-derived FFR values was significantly higher when compared with the risk of individuals with higher values. Specifically, the pooled HR for the lower angiography-derived FFR was 3.98 (95% CI, 2.37–6.66) for target vessel MI (Figure 2E).

### 3.7. The Effect of the Post-PCI Angiography-Derived FFR on TLR

During the follow-up period, the magnitude of risk for TLR in individuals with lower post-PCI angiography-derived FFR values was significantly higher compared with the risk of individuals with higher values. The pooled HR for the lower angiography-derived FFR was 6.27 (95% CI, 3.08–12.79) for TLR (Figure 2F).

### 3.8. Publication Bias

**Figure 3:**
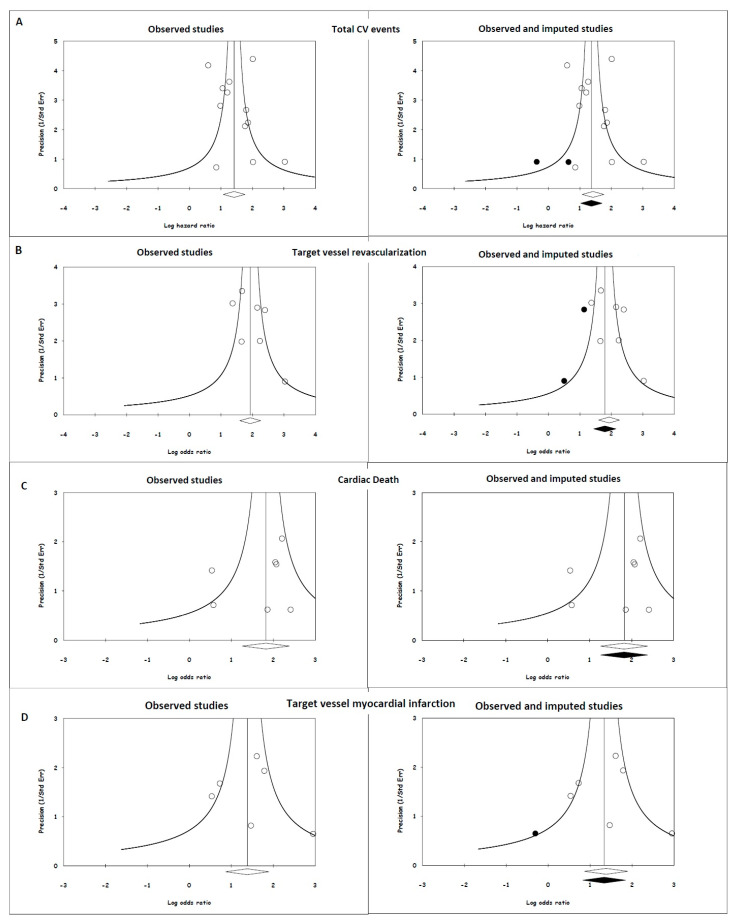
Publication bias for endpoints and their potential effects. (**A**) Total CV events; (**B**) target vessel revascularization; (**C**) cardiac death; and (**D**) target vessel myocardial infarction. The open circles in the left and right plots depict individual studies relating the lower value of the angiography-derived FFR with cardiovascular events, and the open diamonds are the HR and 95% CI for the meta-analysis. The solid circles in the right side of the figure represent imputed studies, and the solid diamonds are the HR and 95% CI for the meta-analysis after adjusting for publication bias.

The funnel plots demonstrate an almost symmetrical distribution of the included studies around the average (Figure 3). The imputed HR values based on the trim-and-fill method were 3.92 (95% CI, 2.79–5.50), 6.01 (95% CI, 4.22–8.56), 6.17 (95% CI, 3.52–10.80) and 3.80 (95% CI, 2.28–6.31) for total CV events, TVR, cardiac death and target vessel MI, respectively, which are not lower than our original risk estimates but are still significant. Regarding the fail-safe N test, the number of missing studies that would need to be added to the analysis to give a statistically nonsignificant overall effect was 497, 282, 50, and 22, respectively. Importantly, it is less likely that there are >41 (497/12 = 41.4), >40 (282/7 = 40.3), 7 (50/7 = 7.1) and 6 (33/6 = 5.5) unpublished studies for every 1 study that we found for total CV events, TVR, cardiac death and target vessel MI, respectively. These findings indicate that the apparent publication bias is inadequate to influence our results or interpretations in a meaningful way.

### 3.9. Meta-Regression Analysis

**Figure 4:**
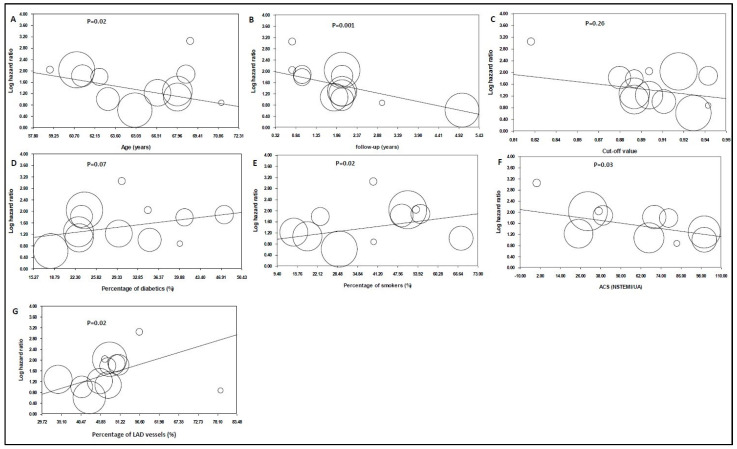
Hazard ratios (HRs) of total cardiovascular events in patients with lower angiography-derived FFR values as an impact of (**A**) age (data from 12 studies [16,17,19,20,22,23,24,25,26,27,28,36]); (**B**) the study’s population follow-up period (data from 12 studies [16,17,19,20,22,23,24,25,26,27,28,36]); (**C**) cut-off value (data from 12 studies [16,17,19,20,22,23,24,25,26,27,28,36]); (**D**) the percentage of the study population with diabetes mellitus (data from 12 studies [16,17,19,20,22,23,24,25,26,27,28,36]); (**E**) the percentage of the study population, who were smokers (data from 11 studies [16,17,19,20,22,24,25,26,27,28,36]); (**F**) the percentage of patients presenting with ACS (data from 10 studies [16,17,18,19,20,21,22,23,24,25]); and (**G**) the percentage of LAD vessels (data from 12 studies [16,17,19,20,21,22,23,24,25,27,28,36]). Each circle represents one study that shows the actual coordinates for that study. The weight of each study is proportional to the size of each circle. The center line shows the values predicted via fixed-effects meta-regression. The vertical axis is on a log scale.

The duration of follow-up was the strongest predictor of the size of the log HR in patients with lower post-PCI angiography-derived FFR values, and it was inversely related to the prognostic role of the post-PCI angiography-derived FFR for total cardiovascular events (*p* = 0.001, Figure 4B). Age at the enrollment indicated inverse associations with the predictive value of the post-PCI angiography-derived FFR value (*p* = 0.02, Figure 4A). The cut-off point of the angiography-derived FFR was not a predictor (*p* = 0.26, Figure 4C), while the percentage of diabetic patients in each study demonstrated a non-statistically significant positive trend (*p* = 0.07, Figure 4D). The percentage of smokers in each study and the percentage of LAD vessels showed positive associations with the predictive role of the post-PCI angiography-derived FFR (*p* = 0.02). The percentage of patients with acute coronary syndrome illustrated a negative association with the predictive value of the angiography-derived FFR after PCI (*p* = 0.03) (Figure 4E–G).

## 4. Discussion

This systematic review and meta-analysis investigated the relationship of the post-PCI angiography-derived FFR with cardiovascular adverse events. We pooled data from thirteen published studies, including approximately 7000 patients who underwent PCI, and investigated adverse outcomes after a mean follow-up period of more than 2 years. Our study is the first meta-analysis to show that lower post-PCI angiography-derived FFR values increase the risk for future cardiovascular adverse events, including TVR, cardiac death, target vessel MI and TLR. Our main finding is that patients with an impaired post-PCI angiography-derived FFR value experienced a four times higher risk of adverse coronary events during the follow-up period. Also, patients with lower post-PCI angiography-derived FFR values presented with seven-, six-, four-, and sixfold higher risks for TVR, cardiac death, target vessel MI and TLR, respectively. According to a sensitivity analysis of three of the included studies, we found that a 0.1 increase in the post-PCI angiography-derived FFR was associated with a risk reduction of 58% for future coronary adverse events. In addition, our study identified that age, clinical presentation and time of follow-up could influence the predictive ability of the post-PCI angiography-derived FFR.

A recent meta-analysis including studies with both post-PCI invasive FFR/iFR and post-PCI angiography-derived FFR values showed that impaired post-PCI physiology assessment values are related to increased adverse cardiac events [38]. Using the same primary outcome as in our study, the authors reported a twofold increase in adverse cardiovascular events in patients with lower post-PCI invasive or angiography-derived FFR values, whereas our study showed a fourfold higher risk in patients with lower angiography-derived FFR values. This difference could be attributed firstly to the inclusion of neutral FFR studies in the meta-analysis by Griffioen et al. [38]. Secondly, in contrast to Griffioen et al. we included two large QFR studies that reported significantly higher risks of adverse cardiovascular events in patients with lower post-PCI QFR values [18,19]. Accordingly, a recent meta-analysis of post-PCI invasive FFR studies reported similar results, supporting the prognostic value of post-PCI physiology [39,40].

The studies used in our meta-analysis included patients with both stable coronary disease and acute coronary syndromes, as well as different clinical scenarios such as in-stent restenosis lesions, indicating that lower post-PCI angiography-derived FFR values are predictive of adverse events in a wide spectrum of coronary artery disease. In most of the included studies, the QFR was used as the method of FFR estimation via angiography [16,17,18,19,20,21,22,23,24,25,26,27,28]. The QFR is a well-studied index of coronary physiology. In a large, randomized trial, QFR-guided PCI showed improved clinical outcomes compared to angiography-guided PCI [10,11]. According to the results of our study, post-PCI guidance using the QFR could offer additional clinical benefits.

The presence of residual ischemia after revascularization can lead to adverse events. Common reasons for residual ischemia are diffuse stenosis beyond the margins of the stent, untreated lesions, or marginal-to-stent coronary artery dissection [24,41]. Intracoronary imaging could be helpful in identifying the underlying pathology. IVUS has shown promising results in revascularization guidance [42,43] and could be used adjacent to coronary physiology indices. Another non-invasive modality that has shown good correlations with the invasive FFR and QFR is fractional flow reserve–computed tomography (FFR-CT), which combines computational fluid dynamics and the coronary artery tree demonstration from coronary computed tomographic angiography [44,45,46,47,48,49]. The prognostic role of the post-PCI FFR-CT needs further investigation.

Microvascular dysfunction predicts adverse cardiac events independently from the fractional flow reserve and successful epicardial coronary revascularization [50]. The index of microcirculatory resistance (IMR) is the gold standard method for coronary microvascular assessment. Based on recent studies, the post-PCI QFR, in either an acute or elective setting, has been incorporated into algorithms for assessing microvascular dysfunction. Many studies have investigated the prognostic role of these angiography-derived indexes of microvascular dysfunction, such as IMRangio and non-hyperaemic IMRangio, providing promising results that are comparable to the ones achieved via the actual measurement of the IMR [51,52,53,54,55]. The additional post-PCI prognostic information that the QFR can offer via post-PCI IMR estimation provides an advantage to this method.

### 4.1. Clinical Implications

The post-PCI angiography-derived FFR had better predictive value in patients with some specific characteristics. These characteristics include a younger age, smoking, presentation with chronic coronary artery disease and coronary artery disease located in the LAD. Also, the post-PCI angiography-derived FFR mainly predicts short-term coronary adverse events because adverse events occurring shortly after the procedure are related to residual stenosis. The cut-off point does not affect its predictive value as long as it ranges from 0.88 to 0.94.

According to previous studies, lower post-PCI QFR values can predict vessel-oriented composite endpoints during follow-up [24]. A higher post-PCI QFR value in patients with three-vessel disease who underwent PCI was associated with a lower risk for vessel-oriented clinical outcomes [25]. Another clinical implication is in patients with in-stent restenosis after DES implantation. Recent studies described that lower QFR values after drug-coated balloon angioplasty were related to worse clinical outcomes during follow-up [16,22]. Patients presenting with acute coronary syndrome may benefit from QFR measurements immediately after the culprit lesion is stented but also from the assessment of non-culprit vessels [20,23].

### 4.2. Strengths and Limitations

A limitation of this meta-analysis is that all studies apart from one were retrospective cohort studies. Also, the currently included studies had small population sizes. However, when combined, the studies provide a substantial sample size for providing conclusive results. Furthermore, clinically relevant outcomes such as heart failure and emergency hospitalizations could not be investigated due to a lack of reporting in the available studies. However, data on revascularization and MI rates imply a higher number of elective and emergency hospitalizations in patients with low post-PCI angiography-derived FFR values.

## 5. Conclusions

Post-PCI angiography-derived FFR assessment is a predictor for cardiovascular events during the follow-up period. When performed in patients with a high risk of recurrent events, such as diabetics, smokers and patients undergoing PCI in the LAD, it could provide an effective tool to estimate patient risk in the catheterization laboratory in a wire-free manner and without any medications to guide both acute (in the catheterization laboratory) and long-term (secondary prevention) management.

## Figures and Tables

**Table 1:** Overview of studies on the association of angiography-derived FFR and cardiovascular events.

First Author. Year (Ref.)	Sample Size	Mean Age ± SD	FU (Years)	Men (%)	LAD (%)	LCX (%)	RCA (%)	Indication	Method	Cut-Off	Outcomes	Results	Adjusted for	NOS
Biscaglia et al., 2019 [24]	602	68	1.8	443 (74.0)	356 (48.0)	184 (24.0)	211 (28.0)	SIHD NSTE-ACS	QAngio XA 3D (Medis Medical Imaging Systems)	≤0.89	VOCE CV death TVMI TVR	Post-PCI QFR was significantly lower in vessels with the vessel-oriented composite endpoint during follow-up compared with those without it.	Diabetes, prior MI and post-PCI diameter stenosis	9
Kogame et al., 2019 [25]	440	66.6	2	364 (92.3)	352 (45.7)	243 (31.5)	176 (22.8)	SIHD NSTE-ACS	QAngio XA 3D (Medis Medical Imaging Systems)	<0.91	VOCE CV death TVMI TVR	The incidence of 2-year VOCE in the vessels with post-PCI QFR <0.91 was significantly higher compared with vessels with post-PCI QFR≥ 0.91.	Creatinine clearance, LAD stenosis and SYNTAX score	9
Tang et al., 2020 [20]	186	63.1	2	140 (75.3)	169 (40.7)	106 (25.5)	140 (33.7)	STEMI	QFR system software (AngioPlus, Pulse Medical Imaging Technology)	≤0.91	VOCE	The multivariable model demonstrated that low post-PCI QFR was an independent predictor of 2-year VOCE.	Diabetes mellitus, culprit lesion, diffuse disease and peak troponin I during the first hospitalization	8
Tang et al., 2021 [22]	177	68.6	1	143 (81.1)	93 (50.3)	37 (20.0)	55 (29.7)	DES-ISR lesions treated with DCB	QFR system software (AngioPlus, Pulse Medical Imaging Technology)	≤0.94	VOCE CV death TVMI TVR	Post-procedural QFR ≤ 0.94 was an independent predictor of 1-year VOCE.	Diabetes mellitus and diameter stenosis (post-procedural in stent)	8
Zhang et al., 2022 [18]	1805	60.9	2	1268 (70.2)	1078 (48.4)	481 (21.6)	663 (30.0)	SIHD ACS	QFR system software (AngioPlus, Pulse Medical Imaging Technology)	≤0.92	VOCE	Post-PCI QFR results ≤0.92 were associated with a higher risk of 2-year VOCE.	No adjustment	9
Dai et al., 2022 [19]	1395	61.3	2	960 (68.8)	857 (50.9)	369 (21.9)	459 (27.2)	SIHD ACS	QAngio XA 3D (Medis Medical Imaging Systems, Leiden, The Netherlands)	<0.89	VOCE	Vessels with low post- PCI QFR demonstrated higher vessel- oriented composite outcome risk after stent implantation.	No adjustment	8
You et al., 2022 [17]	224	71.1	3	152 (67.9)	177 (79.0)	35 (15.6)	12 (5.4)	SIHD ACS	QFR system software (AngioPlus, Pulse Medical Imaging Technology)	≤0.94	TLF	Post-PCI QFR results ≤0.94 was not a predictor of TLF.	No adjustment	7
Liu et al., 2021 [16]	169	62.5	1	128 (75.5)	81 (47.9)	25 (14.8)	63 (37.3)	SIHD NSTE-ACS DES-ISR lesions treated with DCB	QAngio XA 7.3 (Medis Medical Imaging)	≤0.89	VOCE	Post-procedural µQFR ≤ 0.89 was associated with a 6-fold higher risk of VOCE than lesions with µQFR > 0.89.	Diabetes mellitus Difference of DCB diameter and RVD (per 0.10-mm increase)	8
Erbay et al., 2021 [23]	792	68	2	548 (62.9)	691 (34.2)	650 (32.1)	682 (33.7)	ACS	QAngio XA/3D (Medis)	≤0.89	MACE	Independent predictor of major adverse cardiovascular events after ACS.	Age, sex, medical history, type of ACS and, LVEF	9
Cai et al., 2021 [21]	208	63.3	0.75	163 (78.4)	98 (47.1)	32 (15.4)	74 (35.6)	SIHD DES-ISR lesions treated with DCB	QAngio XA/3D (Medis)	≤0.9	ISR	Independently associated with recurrent restenosis after DCB angioplasty.	Vessel caliber, lesion length and diameter stenosis at baseline	7
Neleman et al., 2022 [27]	748	65	5	526 (70.3)	356 (42.8)	NR	NR	SIHD ACS	CAAS Workstation 8.2 (Pie Medical Imaging)	≤0.93	TVF	Lower post-PCI vFFR values are associated with significantly increased risks of TVF and TVR at 5-year follow-up.	No adjustment	7
Suzuki et al., 2019 [26]	45	68.9	1.5–2.5	41 (91)	NR	NR	NR	PCI DES	QAngio XA/3D (Medis)	≤0.82	TVR	Vessel QFR was significantly lower in TVR group.	No adjustment	5
Zhou et al., 2022 [28]	136	59	0.75	91 (66.9)	90 (56.6)	33 (20.8)	36 (22.6)	PCI DES	FLASH ANGIO (Rainmed)	<0.90	TVF	Lower post-PCI caFFR was associated with a higher rate of 9-month TVF.	Age, gender and diabetes mellitus	6

ACS: Acute coronary syndrome; DCB: drug-coated balloon; DES: drug-eluding stent; NSTE-ACS: non-ST elevation acute myocardial infarction; ISR: in stent restenosis; SIHD: stable ischaemic heart disease; STEMI: ST elevation myocardial infarction; TLF: target vessel failure, including target lesion cardiac death (TL-CD), target lesion myocardial infarction (TL-MI) and clinically driven-target lesion revascularization (CD-TLR); TVF: target vessel failure, defined as a composite of target vessel-related myocardial infarction (MI), target vessel-related revascularization (TVR) and cardiac death; VOCE: composite of vessel-related cardiovascular death, vessel-related MI and ischemia-driven target vessel revascularization (TVR); MACE, major adverse cardiovascular event, including all-cause mortality, nonfatal myocardial infarction and ischemia-driven coronary revascularization; NR: not reported; NOS: Newcastle–Ottawa scale.

## Data Availability

The data that support the findings of this meta-analysis are available from the corresponding author upon reasonable request.

## Supplementary Materials

The following supporting information can be downloaded at: https://www.mdpi.com/article/10.3390/jpm13081251/s1. Prisma checklist.
